# ce‐Subpathway: Identification of ceRNA‐mediated subpathways via joint power of ceRNAs and pathway topologies

**DOI:** 10.1111/jcmm.13997

**Published:** 2018-11-12

**Authors:** Chenchen Feng, Chao Song, Ziyu Ning, Bo Ai, Qiuyu Wang, Yong Xu, Meng Li, Xuefeng Bai, Jianmei Zhao, Yuejuan Liu, Xuecang Li, Jian Zhang, Chunquan Li

**Affiliations:** ^1^ School of Medical Informatics Daqing Campus Harbin Medical University Daqing China; ^2^ Department of Pharmacology Daqing Campus Harbin Medical University Daqing China; ^3^ School of Nursing Daqing Campus Harbin Medical University Daqing China; ^4^ The fifth Affiliated Hospital of Harbin Medical University Daqing China

**Keywords:** ceRNAs, differentially expressed genes, subpathways, topological property

## Abstract

Competing endogenous RNAs (ceRNAs) represent a novel mechanism of gene regulation that may mediate key subpathway regions and contribute to the altered activities of pathways. However, the classical methods used to identify pathways fail to specifically consider ceRNAs within the pathways and key regions impacted by them. We proposed a powerful strategy named ce‐Subpathway for the identification of ceRNA‐mediated functional subpathways. It provided an effective level of pathway analysis via integrating ceRNAs, differentially expressed (DE) genes and their key regions within the given pathways. We respectively analysed one pulmonary arterial hypertension (PAH) and one myocardial infarction (MI) data sets and demonstrated that ce‐Subpathway could identify many subpathways whose corresponding entire pathways were ignored by those non‐ceRNA‐mediated pathway identification methods. And these pathways have been well reported to be associated with PAH/MI‐related cardiovascular diseases. Further evidence showed reliability of ceRNA interactions and robustness/reproducibility of the ce‐Subpathway strategy by several data sets of different cancers, including breast cancer, oesophageal cancer and colon cancer. Survival analysis was finally applied to illustrate the clinical application value of the ceRNA‐mediated functional subpathways using another data sets of pancreatic cancer. Comprehensive analyses have shown the power of a joint ceRNAs/DE genes and subpathway strategy based on their topologies.

## 1 INTRODUCTION

MicroRNAs (miRNAs) are small, endogenous, non‐coding RNA molecules that bind to microRNA response elements (MREs) contained in their target mRNAs.^1^ miRNAs are known to target dozens of mRNA transcripts, while mRNAs harbour multiple MREs and thus can be regulated by multiple miRNAs. The fact that distinct RNA molecules can be targeted by common miRNAs leads researchers to suggest the concept of gene regulation by competition for common miRNAs. And those RNA molecules that act as miRNA decoys have been termed as ceRNAs.^2^ Many important transcripts can be reconsidered and functionalized, partly through the identification of competing endogenous mechanism, presenting a framework for the prediction and validation of ceRNAs.^3^ Moreover, large‐scale analyses have shown that ceRNAs play the crucial roles in complex biological processes of many diseases.^4^, ^5^, ^6^ For example, the pseudogene PTENP1 competes with the important tumour suppressor gene PTEN for interaction with miR‐499‐5p, thus regulating PTEN protein levels.^7^ PTEN is known to be frequently disrupted in multiple cancers and governs multiple biological processes, including survival, proliferation and energy metabolism.^8^ As the study of ceRNAs progress, ceRNA interactions are found to work in the pathways. For example, Sumazin et al have constructed glioblastoma‐related ceRNA network and suggested that ceRNAs may function in different regulatory pathways.^9^ However, systematic analysis of competing endogenous mechanism within the pathways is still poorly understood. It is reasonable to expect that some ceRNAs may locate in key regions of the disease‐related pathways or multiple ceRNAs may mediate the same dysregulated regions of the important pathways. Therefore, it is crucial to study ceRNA interactions between the disease‐related genes within the functional pathways and further identify key ceRNA‐mediated subpathway regions, which would provide some clues to the major pathogenesis of human diseases.

Pathway analysis is an effective tool for identifying the pathways or subpathways that are significantly impacted when a biological system is perturbed by stimulation.^10^ However, the current computational approaches applied to pathway identification fail to specifically consider ceRNA interactions within the pathways and key regions impacted by them. For example, hypergeometric test and gene set enrichment analysis (GSEA) approaches have been widely used in pathway analysis.^11^, ^12^ However, they focus only on differentially expressed (DE) genes; ignore those mRNAs that have competing endogenous relationships between each other and the important topologies within the pathways. Another two classical methods, signalling pathway impact analysis (SPIA) and Subpathway‐GM have used pathway topologies for the identification of pathways or subpathways.^13^, ^14^ These methods are excellent in identifying key pathways or subpathway regions, but they still do not consider the effect of competing endogenous mechanism on the pathway or subpathway identification. Recently, although some studies have slightly considered the concept of ceRNAs in pathway analysis, they are short of systematic design, normalized algorithm or available software. For instance, Sun et al have simply imported the interrelated genes within the constructed ceRNA network to some given pathways for annotation, and demonstrated that ceRNAs could potentially modulate multiple signalling pathways.^15^ Obviously, ceRNAs could be the essential components of pathways, the alterations of which may contribute to the altered activities of functional pathways.

In this study, we proposed a novel approach referred to as ce‐Subpathway for the identification of ceRNA‐mediated subpathways. It integrated information from ceRNAs/DE genes, and their key regions within the given pathways to identify significant subpathways associated with the study condition (eg various human diseases). Specifically, ceRNA interactions and disease‐related DE genes were obtained based on gene expression profiles and mapped into the reconstructed pathway graphs. An effective subpathway identification strategy was then applied to locate ceRNA‐mediated functional subpathways from the given pathways. Statistical significance of these subpathways was further evaluated by hypergeometric test, which integrated the impact of the number of both ceRNAs and disease‐related DE genes. The R‐based tool of the ce‐Subpathway strategy has been freely available in GitHub (https://github.com/chunquanlipathway/ce-Subpathway).

## 2 MATERIALS AND METHODS

### 2.1 Gene expression data sets

The study devoted to identifying ceRNA‐mediated functional subpathways associated with various human diseases. The corresponding gene expression profiles were obtained from the NCBI Gene Expression Omnibus (GEO, http://www.ncbi.nlm.nih.gov/geo/) database and TCGA research group (http://tcga-data.nci.nih.gov/), respectively. The following data sets were from GEO: one PAH (GSE33463), one MI (GSE66360), one breast cancer (GSE7562),two oesophageal cancer (GSE20347, GSE74742), two colon cancer (GSE8671, GSE4183) and two pancreatic cancer (GSE32676, GSE57495) data sets. For all the gene expression profiles, the probes were mapped to gene symbol and those probes that mapped to the same gene symbol were merged by averaging their expression values. As for the profiles with raw expression values, gene expression values were log 2 transformed. The following data sets were from TCGA: one breast cancer data set with 849 samples and one pancreatic cancer data set with 177 samples. The processed level 3 RNA‐seq data were downloaded for further analysis.

### 2.2 Obtain mRNA‐related ceRNA interactions and disease‐related DE genes

423 975 miRNA‐mRNA interactions containing 386 miRNAs and 13 802 mRNAs were downloaded from starBase V2.0.^16^ Based on these miRNA‐mRNA interactions, we obtained all the mRNA‐mRNA pairs with sharing the number of common miRNAs ≥3 and estimated their statistical significance by a hypergeometric test. Meanwhile, we required the two mRNAs in each mRNA‐mRNA pair appearing in the same pathway. Those mRNA‐mRNA pairs with hypergeometric test false discovery rate (FDR) adjusted *P* < 0.05 and the two mRNAs of which within the same pathway were retained. According to the theory that the expression of ceRNA interactions was positively correlated, we further computed Pearson correlation coefficient (Pcc) of the above mRNA‐mRNA pairs based on gene expression profiles. Then, all the mRNA‐mRNA pairs with Pcc R >0 and *P* < 0.05 were identified as ceRNA interactions. We obtained these ceRNA interactions, their corresponding *P*‐values of Pcc and all the nonredundant ceRNAs. Here, *P*‐values of Pcc were used to measure the strength of ceRNA interactions.

Differentially expressed genes were the key factor in identifying functional subpathways associated with human diseases. Thus, various diseases‐related DE genes were identified between disease and normal samples based on gene expression profiles, using the significance analysis of microarrays method with a strict cut‐off of FDR adjusted *P* < 0.01.^17^ We obtained disease‐related DE genes, and their corresponding FDR adjusted *P*‐values. Here, FDR adjusted *P*‐values were used to measure DE significance level of disease‐related DE genes.

### 2.3 Map ceRNAs and DE genes into the reconstructed pathway graphs

KGML files (KEGG Markup Language, http://www.genome.jp/kegg/xml/) of the pathways were downloaded from the KEGG database, which provides abundant pathway structure information and is widely used in pathway analysis.^13^, ^18^ These KGML files were converted to list variables in R by applying an R package iSubpathwayMiner.^19^ Specifically, the map nodes were removed from the corresponding KEGG pathway map, and the resulting graphs mainly contained gene products. Two genes were connected by an edge if a common compound was existed in their corresponding reaction in a metabolic pathway, or if they had a relationship such as interaction, binding or modification in a non‐metabolic pathway. Thus, we obtained the reconstructed pathway graphs with the topology structure of each pathway retained.

The disease‐related ceRNAs and DE genes, which we have obtained from the previous step, were then mapped to all the reconstructed pathway graphs. Those ceRNAs or DE genes that could be mapped onto the corresponding nodes of gene products within the reconstructed pathway graphs were defined as key nodes. These key nodes would contribute to identifying important subpathways in the next step.

### 2.4 Locate ceRNA‐mediated subpathways according to key nodes

We located ceRNA‐mediated functional subpathways by integrating the power of a joint ceRNAs/DE genes and subpathway strategy based on their pathway topologies. Three steps were as follows.

First, we developed a computed score named ce‐score, which showed the importance of the DE levels of disease‐related DE genes, the strength of ceRNA interactions and pathway topologies. The formulas were defined as:

(1) $$P^{\mathrm{DE}}=\min\left(P_{i}^{\mathrm{DE}},P_{j}^{\mathrm{DE}}\right)$$

(2) $$P=P^{\mathrm{DE}}\times P^{\mathrm{cor}}$$

(3) $$z=\theta^{-1}\left(1-P\right)$$

(4) $$\text{ce}-\text{score}=\exp(-\frac{d}{z})$$

where $P_{i}^{\mathrm{DE}}$ and $P_{j}^{\mathrm{DE}}$ are the FDR adjusted *P*‐values of key node *i* and *j*, respectively; *P* ^DE^ is the minimum value between $P_{i}^{\mathrm{DE}}$ and $P_{j}^{\mathrm{DE}}$; *P* ^cor^ is the *P*‐value of Pcc between key node *i* and *j*;* z* is the value of the inverse normal cumulative distribution function (θ^−1^) that is converted from *P*;* d* is the length of the shortest path between key node *i* and *j* in any one pathway, calculated by breadth‐first search algorithm; ce‐score is the computed score.

We computed the ce‐scores between any two key nodes in every given pathway graph. The key nodes could be ceRNAs, DE genes or both of them. The smaller FDR adjusted *P*‐value represented more significant DE level of disease‐related DE genes. The smaller *P*‐value of Pcc represented stronger ceRNA interactions. Here, if the relationship between two key nodes was not ceRNA interaction, we set its *P*‐value of Pcc to 1; if one key node was not DE gene, we set its FDR adjusted *P*‐value to 1. Thus, when the nodes had more significant DE level, stronger ceRNA interaction or shorter path length, the ce‐score would be greater. And this would help in identifying more important ceRNA‐mediated functional subpathways. In contrast, when the nodes were not ceRNAs/DE genes or more distance from each other, the ce‐score would be smaller.

Second, we estimated an appropriate threshold ω to screen the greater ce‐scores. Here, we used the empirical probability distribution function. Actually, we ranked all the ce‐scores between any two key nodes within all the reconstructed pathway graphs, and regarded a value which was greater than 75% of the ce‐scores as threshold ω. If the ce‐score between any two key nodes was greater than ω, these two key nodes and other nodes at their shortest path (many other nodes that may or may not be ceRNAs or DE genes) were added to the same node set. This process was recurrent for all key nodes. All the node sets formed the basis for locating subpathways. Flexibility could be introduced through varying threshold ω by users. A larger ω indicated that only key nodes with stronger ceRNA interaction, more significant DE level or shorter path length could be added to the same node set, the identified subpathways would thus form a smaller scale. As the threshold ω decreased, the number of some other nodes except for key nodes within subpathways would increase, and then the scale of subpathways would become larger.

Third, the idea of lenient distance similarity was used to locate ceRNA‐mediated subpathways.^14^, ^20^ According to each node set above, we extracted the corresponding subgraph from pathway graph and defined those subgraphs with the number of nodes ≥5 as ceRNA‐mediated functional subpathways because subgraphs with small scales were only scatter node sets and could not usually form biologically meaningful subpathways.

### 2.5 Evaluate statistical significance of the ceRNA‐mediated subpathways

After locating all the ceRNA‐mediated functional subpathways, we further evaluated their statistical significance by a hypergeometric test. The following values were required: (a) the number of ceRNAs and DE genes submitted for analysis; (b) the number of background genes; (c) the number of ceRNAs and DE genes annotated to each subpathway; and (d) the number of background genes annotated to each subpathway. All human genes in KEGG were considered as background genes. The hypergeometric *P*‐value of statistical significance was defined as:

(5) $$P=1-\sum_{x=0}^{r_{\mathrm{ce}}+r_{\mathrm{DE}}-1}\frac{\left(\begin{array}{c} t\\ x \end{array}\right)\left(\begin{array}{c} m-t\\ n_{\mathrm{ce}}+n_{\mathrm{DE}}-x \end{array}\right)}{\left(\begin{array}{c} m\\ n_{\mathrm{ce}}+n_{\mathrm{DE}} \end{array}\right)}$$

where *m* is the number of genes in the whole genome; n_ce_ (n_DE_) is the total number of ceRNAs (DE genes) submitted for analysis, of which *r*_ce_ (*r*_DE_) is involved in the same subpathway containing *t* genes.

When many subpathways were considered, a high false‐positive discovery rate may be likely to occur. Therefore, we further calculated FDR adjusted *P*‐values using the Benjamini‐Hochberg FDR method.

### 2.6 Survival analysis

To evaluate prognosis performance of the significant ceRNA‐mediated functional subpathways, a risk score model was constructed. The risk score for each patient was calculated according to linear combination of the gene expression values weighted by the regression coefficient from the univariate Cox regression analysis, which was defined as:

(6) $$\text{Risk score}=\sum_{i=1}^{k}\beta_{i}\text{Exp}\left(i\right)$$

where *β*_*i*_ is the Cox regression coefficient of gene *i* from an independent training set; Exp(*i*) is the expression value of gene *i* in a corresponding patient; *k* is the number of testing genes.

The median risk score was used as the cut‐off to classify the training set into high‐risk group and low‐risk group. A Kaplan‐Meier survival analysis was then performed for the two classified groups of patients, and statistic significance was assessed using the log‐rank test with a cut‐off value of *P* < 0.05. The Kaplan‐Meier survival curve was utilized to validate the predicted ability of the k‐gene signature model.

## 3 RESULTS

**Figure 1:**
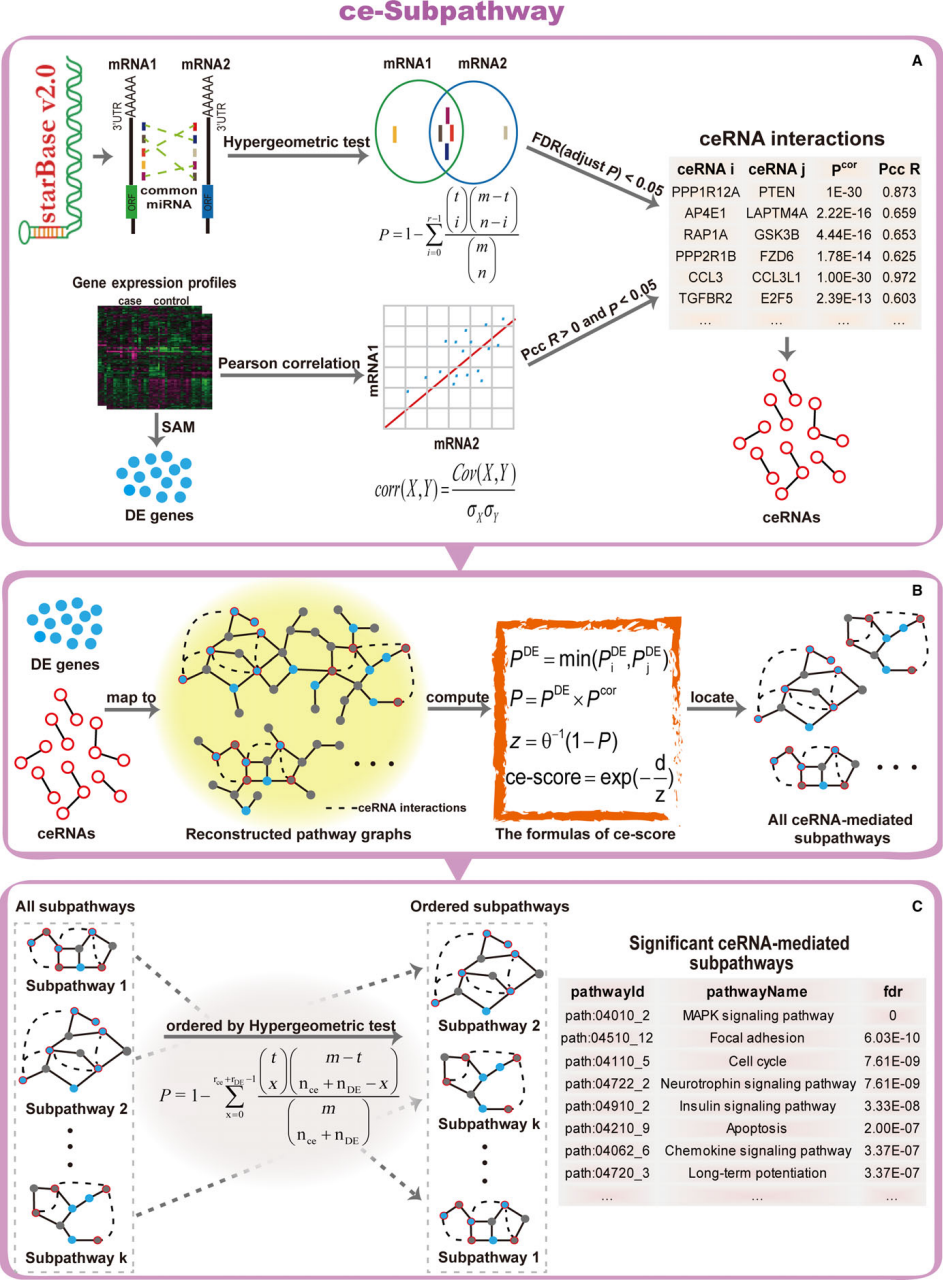
Schematic overview of ce‐Subpathway. A, Obtain mRNA‐related ceRNA interactions and disease‐related DE genes. B, Map ceRNAs and DE genes to the reconstructed pathway graphs and locate all the ceRNA‐mediated subpathways. C, Evaluate statistical significance of the subpathways and identify significant ceRNA‐mediated subpathways

The ce‐Subpathway strategy has been proposed to identify ceRNA‐mediated functional subpathways via a global view of the system‐level integration of ceRNAs, disease‐related DE genes and pathway topologies (Figure 1). In this study, we firstly compared the ce‐Subpathway strategy with four other non‐ceRNA‐mediated pathway/subpathway identification methods at the system level, including hypergeometric test, GSEA, SPIA and Subpathway‐GM. These methods were commonly used for pathway analysis.^11^, ^12^, ^13^, ^14^, ^21^ Then, reliability of ceRNA interactions and robustness/reproducibility of the ce‐Subpathway strategy were validated, respectively. Furthermore, survival analysis was applied to illustrate prognostic value of the ceRNA‐mediated functional subpathways.

### 3.1 PAH‐related ceRNA‐mediated subpathway identification

**Figure 2:**
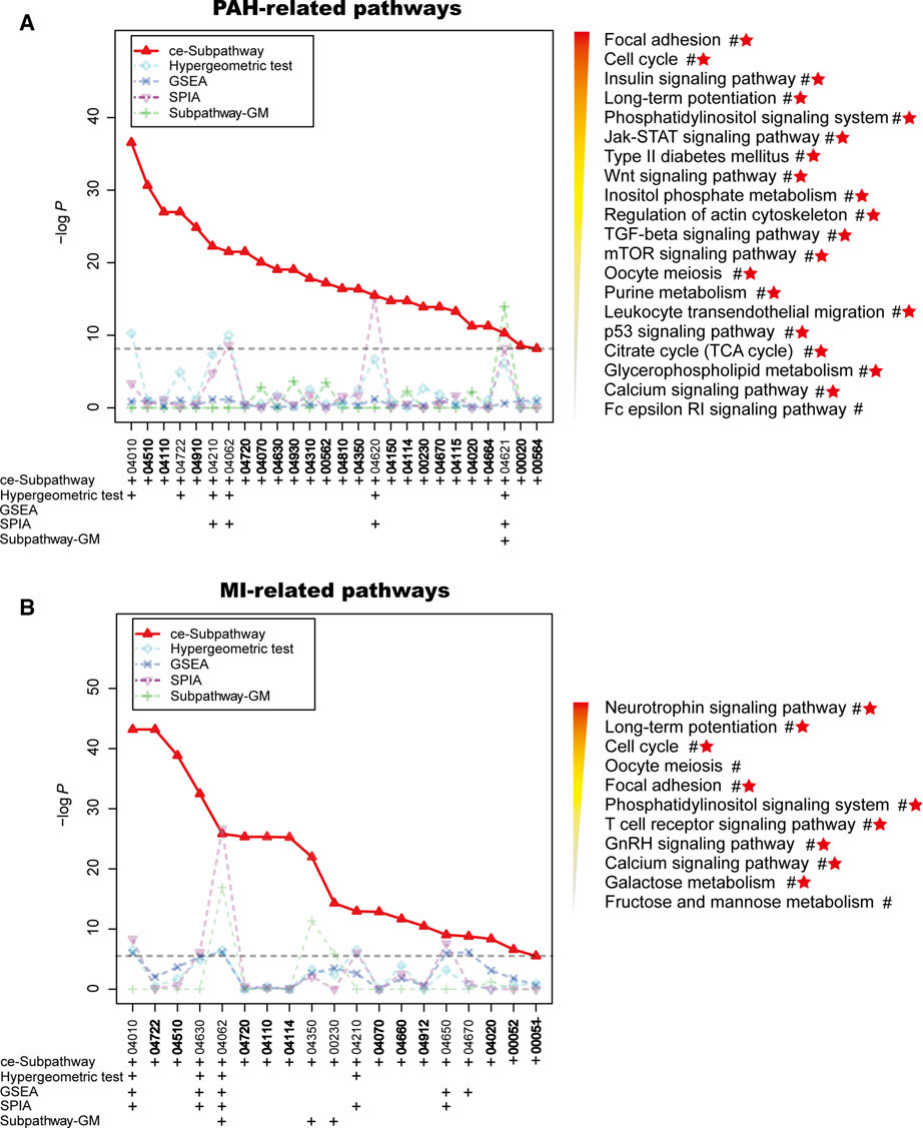
The pathway identification results by different methods. Plots of pathway identification results in PAH (A) and MI (B) data sets according to the ce‐Subpathway, hypergeometric test, gene set enrichment analysis, signalling pathway impact analysis and Subpathway‐GM methods, respectively. Plus sign represents the pathways identified by the corresponding method with a cut‐off of FDR <0.05. Bold labels and the characters near # symbol are the additional pathways uniquely identified by ce‐Subpathway. Pathways that have been well reported to be associated with PAH/MI by curated literatures are marked with red star

The PAH data set (GSE33463, Table 1) was chosen to illustrate the effectiveness of the ce‐Subpathway method. With FDR adjusted *P* < 0.05, we identified 31 significant ceRNA‐mediated functional subpathways from all the reconstructed pathway graphs, which corresponded to 26 entire pathways (Tables 2, S1 and S2). Lots of these pathways were ignored by hypergeometric test, GSEA, SPIA or Subpathway‐GM (Figure 2A, Tables 2 and S2). Hypergeometric test found 25 significant pathways with FDR adjusted *P* < 0.05, but 20 (76.92%) significant pathways identified by ce‐Subpathway could not be detected by hypergeometric test (Figure 2A, Table 2). GSEA identified only four significant pathways with FDR adjusted *P* < 0.05, which were not the same as any significant pathways identified by ce‐Subpathway (Figure 2A, Table 2). The power of SPIA also seemed to be limited, 22 (84.62%) significant pathways identified by ce‐Subpathway were ignored by SPIA (Figure 2A, Table 2). The subpathway identification strategy, Subpathway‐GM, identified lots of subpathways. However, up to 30 significant subpathways corresponding to 25 (96.15%) entire pathways identified by ce‐Subpathway were undetected by Subpathway‐GM (Figure 2A, Table 2). Surprisingly, up to 24 significant subpathways identified by ce‐Subpathway, which corresponded to 20 entire pathways, were simultaneously ignored by the four non‐ceRNA‐mediated methods (Figure 2A, Table 2). In addition, we found that up to 25 pathways identified by ce‐Subpathway have been well reported to be associated with PAH‐related cardiovascular disease (Tables 2 and S2). Only one pathway could not be validated effectively with appropriate references. However, for example, in 25 significant pathways identified by hypergeometric test, up to three pathways were not reported to be associated with PAH by curated literatures (Table S2).

**Table 1:** The data sets used for ceRNA‐mediated subpathway identification

Disease	Data set	No. of disease samples	No. of normal samples	No. of DE genes	No. of ceRNAs	No. of ceRNA interactions
PAH	GSE33463	80	41	2833	950	2135
MI	GSE66360	49	50	1754	822	1492
Breast Cancer	The Cancer Genome Atlas data	762	87	14 657	921	1892

**Table 2:** The significant subpathways identified by ce‐Subpathway using PAH data set

Subpathway ID	PathwayName	ce‐Subpathway	Hypergeometric	Gene set enrichment analysis	SPIA	Subpathway‐GM	Reference(PMID)
path:04010_2	MAPK signalling pathway	0	0.0054	—	—	—	27688788; 28055284
path:04510_12^#^	Focal adhesion	6.03E‐10	—	—	—	—	22293597; 28077433; 27274622
path:04110_5^#^	Cell cycle	7.61E‐09	—	—	—	—	27470556; 27581840; 26273643
path:04722_2	Neurotrophin signalling pathway	7.61E‐09	0.0093	—	—	—	24462831
path:04910_2^#^	Insulin signalling pathway	3.33E‐08	—	—	—	—	26254106; 25921925
path:04210_9	Apoptosis	2.00E‐07	0.0310	—	0.0368	—	28068653; 28036116
path:04062_6	Chemokine signalling pathway	3.37E‐07	0.0339	—	0.0026	—	28393260; 28774332
path:04720_3^#^	Long‐term potentiation	3.37E‐07	—	—	—	—	18704488
path:04070_1^#^	Phosphatidylinositol signalling system	9.24E‐07	—	—	—	—	24084215; 23220709; 29074487
path:04630_2^#^	Jak‐STAT signalling pathway	1.85E‐06	—	—	—	—	24058777; 24058763; 28393260
path:04930_1^#^	Type II diabetes mellitus	1.85E‐06	—	—	—	—	16304314; 23348820
path:04110_3^#^	Cell cycle	3.81E‐06	—	—	—	—	27470556; 27581840; 26273643
path:04310_20^#^	Wnt signalling pathway	4.32E‐06	—	—	—	—	27188753; 26860892
path:00562_1^#^	Inositol phosphate metabolism	6.68E‐06	—	—	—	—	9847264; 15838259; 23077657
path:04630_6^#^	Jak‐STAT signalling pathway	9.18E‐06	—	—	—	—	24058777; 24058763; 28393260
path:04810_5^#^	Regulation of actin cytoskeleton	1.15E‐05	—	—	—	—	24283363; 19188659; 29473816
path:04350_21^#^	TGF‐*β* signalling pathway	1.20E‐05	—	—	—	—	24956016; 18202349
path:04620_14	Toll‐like receptor signalling pathway	2.19E‐05	2.62E‐05	—	2.73E‐05	—	27418552; 26418144; 27712004
path:04150_3^#^	mTOR signalling pathway	3.67E‐05	—	—	—	—	27258250; 26409044
path:04810_19^#^	Regulation of actin cytoskeleton	3.71E‐05	—	—	—	—	24283363; 19188659; 29473816
path:04114_1^#^	Oocyte meiosis	3.71E‐05	—	—	—	—	20886366
path:04670_7^#^	Leucocyte transendothelial migration	6.64E‐05	—	—	—	—	25909334; 25722443
path:00230_8^#^	Purine metabolism	6.64E‐05	—	—	—	—	24656288
path:04115_5^#^	p53 signalling pathway	0.0001	—	—	—	—	27063355; 25290246
path:04020_1^#^	Calcium signalling pathway	0.0004	—	—	—	—	23300272; 15838259; 24770445; 23739180
path:04664_4^#^	Fc epsilon RI signalling pathway	0.0004	—	—	—	—	na
path:04510_10^#^	Focal adhesion	0.0008	—	—	—	—	22293597; 28077433; 27274622
path:04621_1	NOD‐like receptor signalling pathway	0.0008	0.0009	—	0.0034	6.42E‐05	24736319
path:00020_2^#^	Citrate cycle (TCA cycle)	0.0026	—	—	—	—	23964055; 24533144
path:04010_12	MAPK signalling pathway	0.0031	0.0054	—	—	—	27688788; 28055284
path:00564_13^#^	Glycerophospholipid metabolism	0.0035	—	—	—	—	26675529

Subpathways with ^#^ symbol are uniquely identified by ce‐Subpathway. The table lists FDR adjusted *P*‐values.

**Figure 3:**
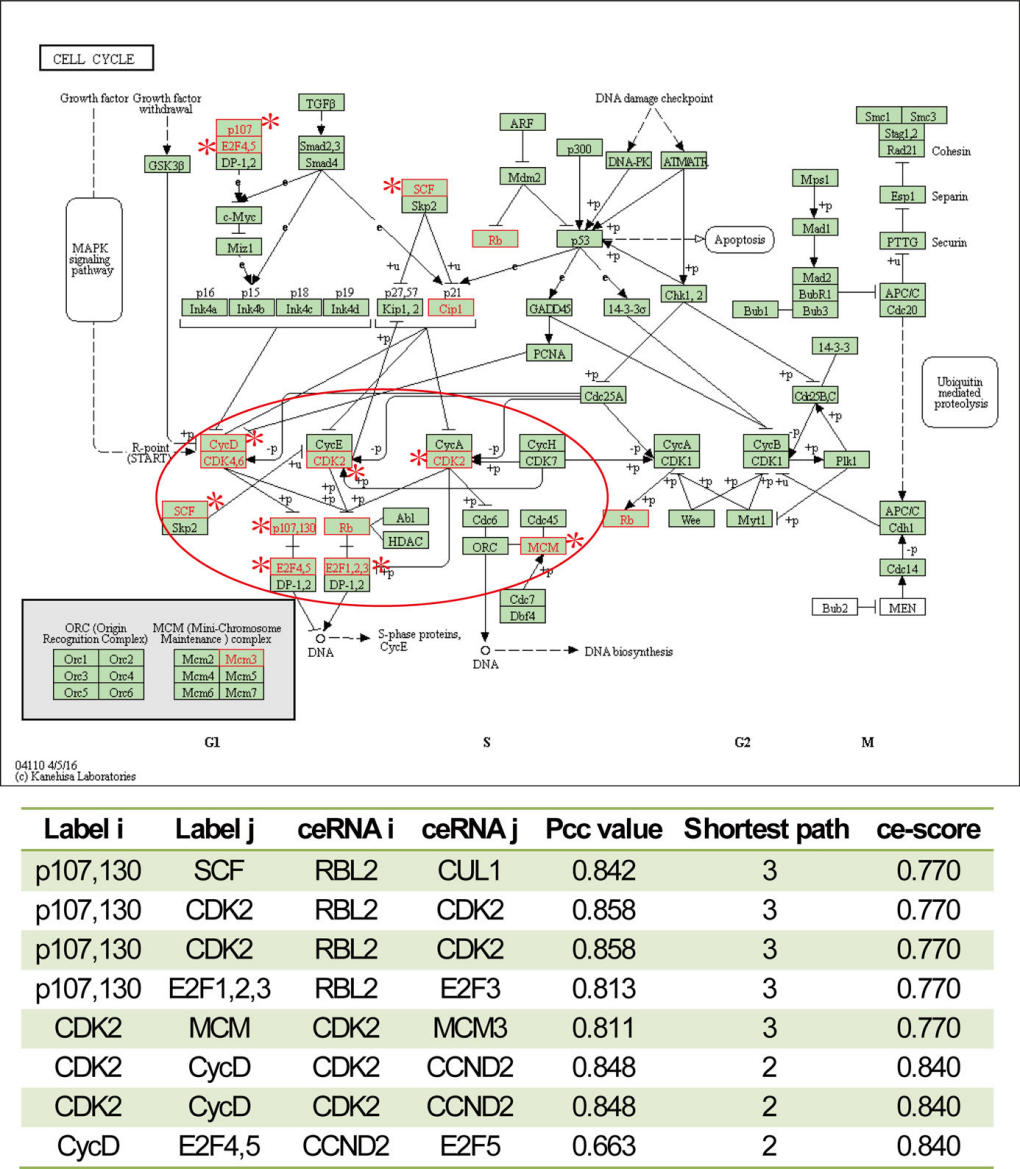
The cell cycle pathway uniquely identified by ce‐Subpathway. The upper figure is the cell cycle pathway in KEGG. Red node labels and borders represent ceRNAs or DE genes mapped to the pathway. Nodes near asterisk symbol belong to the subpathway (path:04110_5) identified by ce‐Subpathway. Key subpathway region is shown in red ellipse. The bottom table lists all the ceRNA interactions of this subpathway with the corresponding ce‐scores. The same row of the table means more than one shortest paths between the ceRNA interaction pair

We focused on a significant ceRNA‐mediated subpathway (path:04110_5), which belonged to “cell cycle” (Figure 3). It was reported to play key roles in the smooth muscle cell proliferation and vascular remodelling.^22^, ^23^ The ce‐Subpathway strategy yielded a FDR adjusted *P*‐value of 7.61E‐09, but cell cycle was not considered as significant in the hypergeometric test, GSEA, SPIA or Subpathway‐GM method (FDR adjusted *P* > 0.4). The key subpathway region was at the bottom of the pathway. As the crucial component of this key subpathway region, CDK2 has been demonstrated to suppress the vascular smooth muscle cell proliferation.^24^ When using the drug “Mevastatin” to treat the pulmonary artery smooth muscle cells, CDK2 was found to show decreased activities.^25^ More importantly, several other key nodes, such as MCM3 and RBL2, respectively had strong ceRNA interactions with CDK2. Some research has indicated that phosphorylation of MCM3 by CDK2 could regulate its function in cell cycle.^26^ In addition, the expression of RBL2 has a high correlation with the growth of vascular endothelial cells.^27^ Strong ceRNA interaction was also found between RBL2 and E2F3. E2F3 has been reported as an important transcription factor that controls proliferation of vascular smooth muscle cells.^28^ The above results suggest that stronger ceRNA interactions, shorter path length and greater ce‐scores are the necessary factors in identifying more important ceRNA‐mediated functional subpathways.

**Figure 4:**
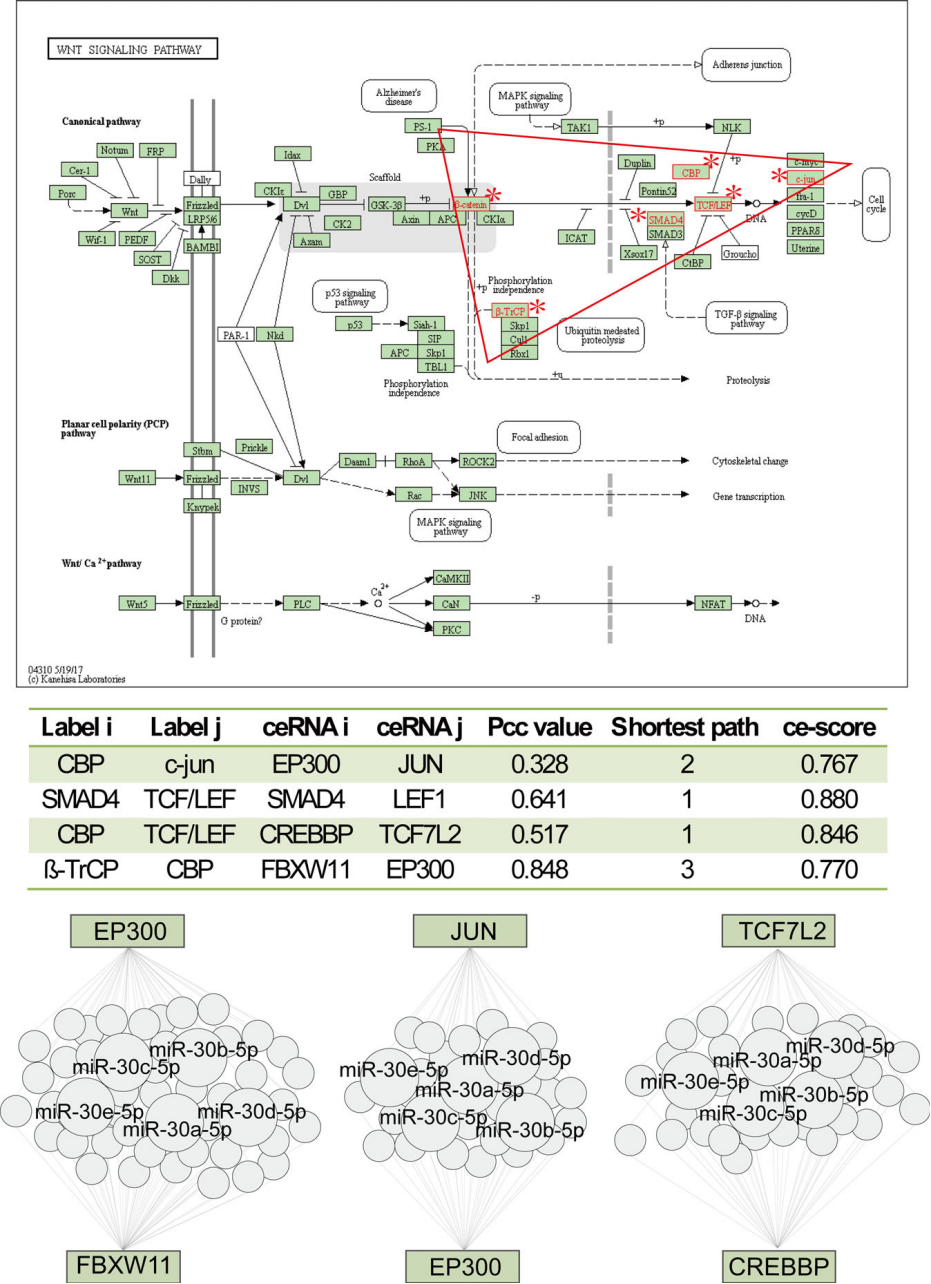
The Wnt signalling pathway uniquely identified by ce‐Subpathway. The upper figure is the Wnt signalling pathway in KEGG. Red node labels and borders near asterisk symbol belong to the subpathway (path:04310_20) identified by ce‐Subpathway. Key subpathway region is shown in red triangle. The middle table lists all the ceRNA interactions of this subpathway with the corresponding ce‐scores. The bottom region shows three pairs of ceRNA interactions in this subpathway, which are formed by competing the common miRNAs

Another ceRNA‐mediated functional subpathway (path:04310_20) belonged to “Wnt signalling pathway” (Figure 4), which has been verified as a crucial regulatory mechanism in PAH. The ce‐Subpathway method yielded a FDR adjusted *P*‐value of 4.32E‐06. Key subpathway identified was located in the canonical pathway region of Wnt signalling pathway. β‐catenin, one of the key nodes in this subpathway, was located in the central position of the canonical pathway region. Activation of β‐catenin would disturb the growth of normal pulmonary arterial smooth muscle cells, but promotes the malignant proliferation.^29^ In the level of miRNAs that mediated EP300 and JUN, CREBBP and TCF7L2, FBXW11 and EP300 ceRNA interactions, we found miR‐30 family was extracted, simultaneously. It has been demonstrated that the miR‐30 family has been a crucial regulator that exerts functions in human pulmonary endothelial cells.^30^ Previous studies have also shown that all genes associated with these ceRNA interactions are implicated in the pathological processes of PAH. For example, EP300 functions as histone acetyltransferase to regulate transcription of genes via chromatin remodelling, overexpression of which could alter the expression levels of ECM proteins and VEGF in endothelial cells.^31^ In a word, both the common miRNAs and ceRNAs of the key ceRNA‐mediated subpathways have been suggested to be closely associated with PAH.

### 3.2 MI‐related ceRNA‐mediated subpathway identification

The MI data set (GSE66360, Table 1) was used to demonstrate the reliability of ce‐Subpathway in identifying specific subpathways associated with different disease subtypes. With a strict cut‐off of FDR adjusted *P* < 0.05, 22 significant ceRNA‐mediated subpathways were identified from all the reconstructed pathway graphs (Tables S1 and S2). They corresponded to 19 entire pathways, up to 89.47% (17/19) of which were well reported to be associated with MI‐related cardiovascular disease (Figure 2B, Tables 3 and S2). Many pathways identified by ce‐Subpathway were not detected by hypergeometric test, GSEA, SPIA or Subpathway‐GM method (Figure 2B, Tables 3 and S2). Specifically, these four methods found 16, 44, 23 and 62 significant pathways or subpathways with FDR adjusted *P* < 0.05, respectively. However, they ignored 15 (78.95%), 14 (73.68%), 14 (73.68%) and 16 (84.21%) significant pathways identified by ce‐Subpathway, respectively (Figure 2B, Table 3). What is s more, 11 significant ceRNA‐mediated subpathways identified by ce‐Subpathway were simultaneously undetected by the four non‐ceRNA‐mediated methods, corresponding to 11 entire pathways (Figure 2B, Table 3).

**Table 3:** The significant subpathways identified by ce‐Subpathway using MI data set

Subpathway ID	PathwayName	ce‐Subpathway	Hypergeometric	Gene set enrichment analysis	Signalling pathway impact analysis	Subpathway‐GM	Reference(PMID)
path:04010_2	MAPK signalling pathway	0	0.0108	0.0153	0.0030	—	27538767; 23264165
path:04722_6^#^	Neurotrophin signalling pathway	0	—	—	—	—	20122881; 23831387
path:04510_1	Focal adhesion	2.01E‐12	—	—	—	0.0226	27825850; 26330161
path:04630_5	Jak‐STAT signalling pathway	1.66E‐10	0.0347	0.0235	0.0141	—	15723072; 23128561; 22749532
path:04062_1	Chemokine signalling pathway	1.67E‐08	0.0112	0.0142	9.46E‐09	8.25E‐06	26264282; 15322539; 29933226
path:04720_2^#^	Long‐term potentiation	2.38E‐08	—	—	—	—	15019859; 24361546
path:04110_4^#^	Cell cycle	2.38E‐08	—	—	—	—	18508765; 25904597
path:04114_1^#^	Oocyte meiosis	2.50E‐08	—	—	—	—	NA
path:04510_2^#^	Focal adhesion	1.64E‐07	—	—	—	—	27825850; 26330161
path:04350_2	TGF‐*β* signalling pathway	2.42E‐07	—	—	—	0.0003	28446968; 27614871
path:00230_1	Purine metabolism	4.95E‐05	—	—	—	0.0167	25015064
path:04010_3	MAPK signalling pathway	5.10E‐05	0.0108	0.0153	0.0030	—	27538767; 23264165
path:04210_16	Apoptosis	0.0001	0.0108	—	0.0150	—	28602551; 25304741
path:04070_6^#^	Phosphatidylinositol signalling system	0.0001	—	—	—	—	18679782; 11940366
path:04660_9^#^	T cell receptor signalling pathway	0.0003	—	—	—	—	27213032; 26646702
path:04912_3^#^	GnRH signalling pathway	0.0007	—	—	—	—	26264282
path:04650_13	Natural killer cell mediated cytotoxicity	0.0019	—	0.0157	0.0051	—	26725916; 21388427
path:04010_32	MAPK signalling pathway	0.0022	0.0108	0.0153	0.0030	—	27538767; 23264165
path:04670_12	Leucocyte transendothelial migration	0.0022	—	0.0143	—	—	23642836; 29845217
path:04020_1^#^	Calcium signalling pathway	0.0030	—	—	—	—	26067684; 29758552
path:00052_6^#^	Galactose metabolism	0.0103	—	—	—	—	26498380; 22803435
path:00051_5^#^	Fructose and mannose metabolism	0.0217	—	—	—	—	NA

Subpathways with ^#^ symbol are uniquely identified by ce‐Subpathway. The table lists FDR adjusted *P*‐values.

**Figure 5:**
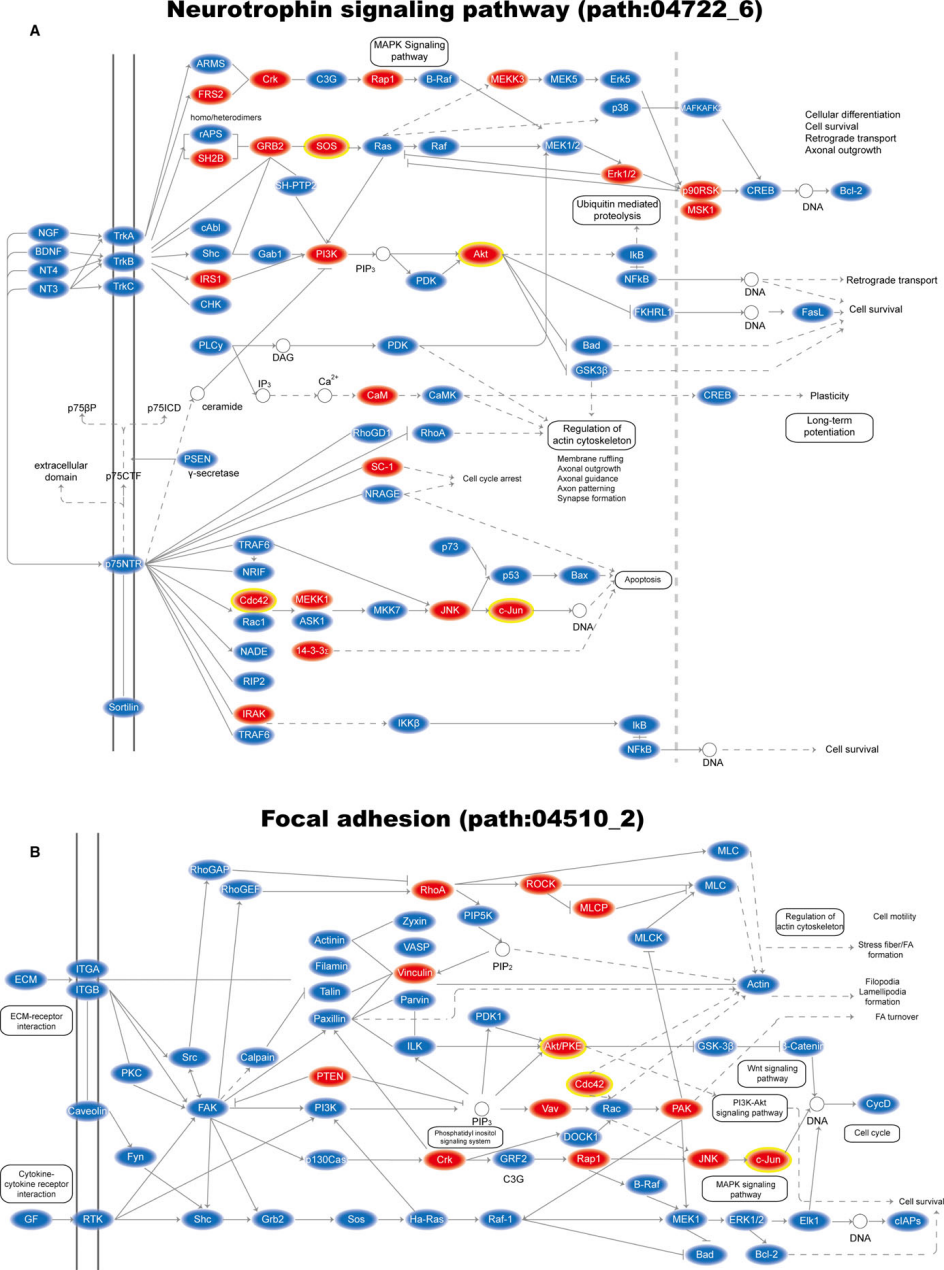
The MI‐related subpathways where key nodes are annotated. A, Plot of key MI‐related subpathway (path:04722_6) belongs to neurotrophin signalling pathway; B, Plot of key MI‐related subpathway (path:04510_2) belongs to Focal adhesion. Key ceRNAs are shown with red ellipse; key DE genes are shown with yellow border

The most significant of these additional ceRNA‐mediated subpathways (path:04722_6) belonged to the “neurotrophin signalling pathway,” which has been demonstrated to be involved in the regulatory processes of injury of MI.^32^ We extracted the topological structure of this subpathway and found numerous MI‐related DE genes and ceRNAs (Figure 5A). For example, DE genes c‐Jun and Cdc42 had a strong ceRNA interaction relationship. Some research has shown that Cdc42 could stimulate the activity of Jun kinase 1 and further mediate transcriptional regulation.^33^, ^34^ Inhibition of N‐terminal kinase of Jun would decrease cardiomyocyte apoptosis and infarct size after myocardial ischaemia and reperfusion.^35^ The other additional ceRNA‐mediated subpathway (path:04510_2) belonged to “focal adhesion” with a FDR of 1.64E‐07. The famous ceRNA PTEN was found to implicate in ceRNA interactions of this subpathway (Figure 5B). Studies have reported that PTEN is critically involved in post‐MI remodelling, the expression level of which is regulated in infarcted heart.^36^

Interestingly, by comparing PAH‐related and MI‐related ceRNA‐mediated subpathways, we found that several corresponding pathways of them were the same, though only a small fraction of disease‐related DE genes were overlapped. These pathways included mitogen‐activated protein kinase (MAPK) signalling pathway, Jak‐STAT signalling pathway and cell cycle. But it is also worth noting that different subpathway regions were located. For example, PAH‐related subpathways were located in Jak‐STAT signalling pathway (path:04630_2 and path:04630_6), but MI‐related subpathway was located in Jak‐STAT signalling pathway (path:04630_5). More information could be seen in Tables 2 and 3. Actually, PAH and MI are the different subtypes of cardiovascular disease that are composed of more than 10 disease subtypes. Some studies have found the close correlations in different subtypes of cardiovascular disease. For example, sustained cardiac hypertrophy and PAH could result in heart failure^37^, ^38^; coronary artery disease could induce the injury of cardiac ischaemic and then lead to MI.^39^ There may be similar molecular mechanisms but different pathogenic factors between the different subtypes of cardiovascular disease. These results demonstrate the importance of ce‐Subpathway in investigating the similar or specific features across various disease subtypes.

### 3.3 Cancer‐related ceRNA‐mediated subpathway identification

**Figure 6:**
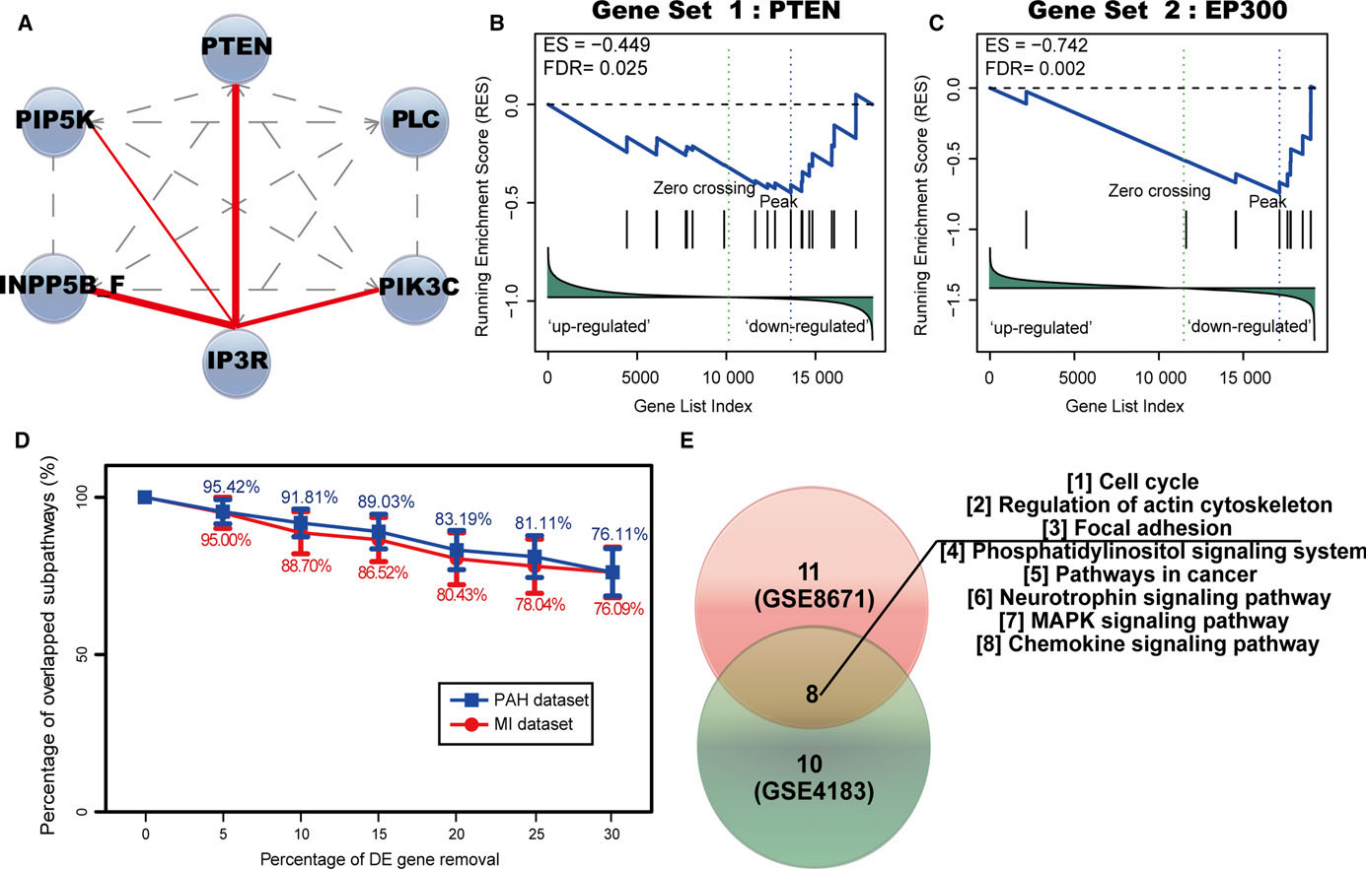
Results of reliability validation and robustness/reproducibility analysis. (A) Plot of key breast cancer‐related subpathway (path:04070_8) belongs to Phosphatidylinositol signalling system. Key nodes are shown with blue nodes; the direct interactions in the pathway between two genes are shown with gay dashed lines; the ceRNA interactions are shown with red solid lines, edge width is proportional to the ce‐score. Results of reliability validation of ceRNA interactions used in the study based on PTEN (B) and EP300 (C) gene knockdown profiles with a cut‐off of FDR <0.05. (D) Results of robustness analysis of the ce‐Subpathway method. PAH data set is shown with blue broken line; MI data set is shown with red broken line. (E) Results of reproducibility analysis of the ce‐Subpathway method. The numbers of significant pathways identified in two different colon cancer data sets are shown with pie charts of different colours

Our challenge not only lies in obtaining biologically meaningful subpathways, but also in interpreting the reliability of ceRNA interactions used in the study. Based on the breast cancer data set from The Cancer Genome Atlas (TCGA; Table 1), we identified 16 significant ceRNA‐mediated subpathways by ce‐Subpathway. They corresponded to 12 entire pathways, which were well associated with breast cancer (Table 4). Such as phosphatidylinositol signalling system, which is critical to normal and malignant cellular processes, including proliferation, apoptosis and metabolism.^40^ Studies have shown that mutations in genes that constitute the phosphatidylinositol 3‐kinase (PI3K)‐related pathway occur in >70% of breast cancers.^41^ Clinical and experimental evidence suggests that PI3K signalling activation promotes resistance to some of the current breast cancer therapies.^42^ By extracting topology structure, the famous ceRNA PTEN was found in the significant subpathway region (path:04070_8) of phosphatidylinositol signalling system (Figure 6A). We then took PTEN‐related ceRNAs for reliability validation of ceRNA interaction pairs. Specifically, all the nonredundant ceRNAs associated with PTEN were collected from the corresponding ceRNA interactions as a gene set S. Based on one PTEN gene knockdown profile of breast cancer cell line (GSE7562), lists of genes ranked according to their values of fold change, from the most up‐regulated (at the top of the list) to the most down‐regulated (at the bottom of the list), which was as a background gene list L. Actually, when PTEN was knockdown, PTEN‐related ceRNAs should be expression down‐regulated because ceRNA interaction pairs were positively correlated. Therefore, GSEA was applied to determine whether members of the gene set S tended to occur towards the bottom of the background gene list L, in which case the gene set was correlated with the phenotypic class distinction. We calculated an enrichment score (ES) that reflected the degree to which the set S was overrepresented at the bottom of the entire ranked list L and estimated statistical significance of the ES by using an empirical phenotype‐based permutation test. As a result, PTEN‐related ceRNAs showed the tendency of expression down‐regulated with a FDR adjusted *P*‐value of 0.025 (Figure 6B).

**Table 4:** The significant subpathways identified by ce‐Subpathway using breast cancer data set

Subpathway ID	PathwayName	ce‐Subpathway (FDR)	Reference(PMID)
path:04630_6	Jak‐STAT signalling pathway	4.79E‐06	25104439; 30022447; 29383118
path:04810_7	Regulation of actin cytoskeleton	3.07E‐05	23153535; 23775624
path:04510_1	Focal adhesion	3.13E‐05	24491810; 25631868
path:04510_6	Focal adhesion	3.13E‐05	24491810; 25631868
path:04310_19	Wnt signalling pathway	0.0002	24606421; 25955111; 26129710
path:05215_23	Prostate cancer	0.0002	25421124
path:04010_11	MAPK signalling pathway	0.0003	24882719; 25066297
path:04110_23	Cell cycle	0.0004	25064703; 24369047; 25407488
path:04722_7	Neurotrophin signalling pathway	0.0004	28446206; 27467251
path:00240_13	Pyrimidine metabolism	0.0009	29614418
path:04630_2	Jak‐STAT signalling pathway	0.0012	25104439; 30022447; 29383118
path:04350_26	TGF‐*β* signalling pathway	0.0106	25823021; 25217524; 26223118
path:04510_24	Focal adhesion	0.0106	24491810; 25631868
path:04530_3	Tight junction	0.0106	23934616; 29719617
path:04010_10	MAPK signalling pathway	0.0154	24882719; 25066297
path:04070_8	Phosphatidylinositol signalling system	0.0155	24774538; 25544707
path:05220_25	Chronic myeloid leukaemia	0.0547	18192121
path:04114_13	Oocyte meiosis	0.0574	28849078; 26804550

The similar steps were also performed on EP300‐related ceRNAs. Based on one oesophageal cancer data set from GEO (GSE20347), five ceRNA‐mediated subpathways were identified by ce‐Subpathway. With a FDR adjusted *P*‐value of 0.002, EP300‐related ceRNAs showed the tendency of expression down‐regulated according to one EP300 gene knockdown profile of oesophageal cancer cell line (GSE74742) (Figure 6C). The ceRNA interactions used in the study have been obtained according to rigorous calculation process; here, the reliability is further validated based on gene knockdown profiles of their corresponding ceRNAs.

### 3.4 Robustness and reproducibility analysis of ce‐Subpathway

Within the reconstructed pathway graphs, the disease‐related DE genes and ceRNAs have been mapped to the corresponding nodes of gene products. Those DE gene nodes and ceRNA interactions were the necessary components for retaining the topology structure of each pathway. When some DE genes were removed, ceRNA interactions between two nodes would be broken to varying degrees. Then, robustness of the ce‐Subpathway method was tested by performing data removal tests using PAH‐related or MI‐related DE genes, respectively. To each set of DE genes, we removed the number of DE genes from 5% to 30% at 5% intervals with the remaining DE genes as new input, and repeated the ce‐Subpathway method 20 times for each removal. In the PAH data set, the number of overlapped significant subpathways fell slowly compared with the original data, and the ratio of overlapped subpathways to original significant subpathways remained 76.11% even after removal of up to 30% DE genes (Figure 6D).The similar results were also obtained in the MI data set (Figure 6D). These results indicate that the ce‐Subpathway method is robust to data removal.

To test the reproducibility of the results across different data sets, the ce‐Subpathway method was applied to another two independent colon cancer data sets from GEO (GSE8671, GSE4183). About 11 and 10 significant ceRNA‐mediated subpathways were identified, corresponding to 10 and 9 entire pathways, respectively. Eight pathways were found highly reproducible across these results (Figure 6E), all of which have been reported to be associated with the occurrence and development of colon cancer. For example, focal adhesion kinase signalling that is overexpressed in metastatic colon cancer plays a key role in angiogenesis, cell proliferation and survival, motility and invasion.^43^ The insulin‐like growth factor‐1 receptor tyrosine kinase signalling through the MAPK and phosphatidylinositol 3‐kinase pathways plays a part in transformation and colon tumourigenesis.^44^ The actin‐cytoskeleton pathway is the backbone of cells that allows migration and mobility of cells within the body, which has already been implicated in the development and pathogenesis of invasive metastatic colon cancer.^45^ These results show the power of reproducibility of the ce‐Subpathway method.

### 3.5 Clinical application test of the ceRNA‐mediated subpathways

**Figure 7:**
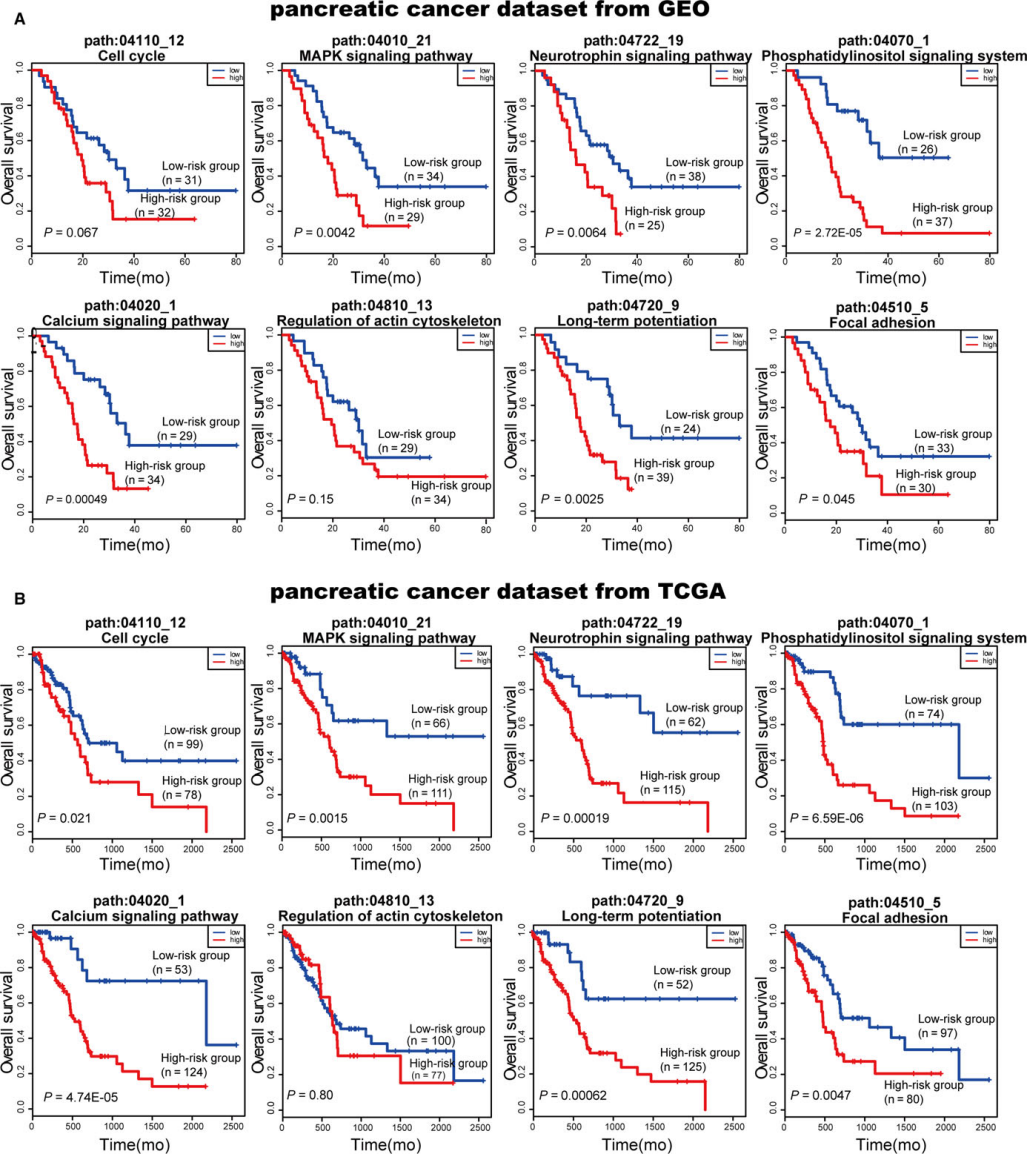
Results of survival analysis of the ceRNA‐mediated subpathways. Survival curves plotted based on two independent pancreatic cancer data sets with survival information: one from GEO (A) and one from The Cancer Genome Atlas (B)

Cancer survival analysis is an essential indicator for effective early detection and improvement in cancer treatment. To further test the clinical application ability of the ceRNA‐mediated functional subpathways, we focused on pancreatic cancer for performing survival analysis. Based on one pancreatic cancer data set from GEO (GSE32676), eight significant ceRNA‐mediated subpathways were identified by performing the ce‐Subpathway method. The genes in each of these significant subpathways were identified as a k‐gene signature. Then, another two independent pancreatic cancer data sets with gene expression and clinical data were obtained from GEO (GSE57495) and TCGA as independent training sets for the risk scores, respectively (see Section 2.2). For instance, the 28 genes in the significant subpathway (path:04010_21, FDR adjusted *P* = 3.32E‐05) of MAPK signalling pathway were identified as a 28‐gene signature. With the 28‐gene signature, patients of the independent training set from GEO (GSE57495) were divided into a high‐risk group (n = 29) and a low‐risk group (n = 34). Patients in high‐risk group had significantly shorter overall survival than those in low‐risk group (*P* = 0.0042; Figure 7A, the second plot). The same model and criteria classified 111 and 66 patients of the other independent training set from TCGA into high‐risk and low‐risk groups, respectively. The overall survival time of high‐risk group patients was also significantly shorter than that of low‐risk group patients (*P* = 0.0015; Figure 7B, the second plot). Actually, the significant results could be observed in six and seven of the eight subpathways based on the two independent pancreatic cancer data sets from GEO and TCGA, respectively (Figure 7A,B). Most importantly, the same six subpathways significantly distinguished the high‐risk and low‐risk groups in both the GEO and the TCGA data sets (Table S3). These subpathways were corresponding to MAPK signalling pathway, neurotrophin signalling pathway, phosphatidylinositol signalling system, calcium signalling pathway, long‐term potentiation and focal adhesion. It is reported that novel agents targeting dysregulated MAPK signalling pathways are being explored in clinical trials as monotherapy or in combination with cytotoxic chemotherapy for pancreatic cancer.^46^ Systematic pathway enrichment analysis of a genome‐wide association study on cancer survival has also revealed an influence of genes involved in cell adhesion and calcium signalling on the patients’ clinical outcome.^47^

To further illustrate the overall discriminatory power of the ceRNA‐mediated subpathways, every gene of the six common subpathways was identified as a single‐gene signature for survival analysis. According to the independent training set from GEO (GSE57495), almost all single‐gene signatures could not distinguish the high‐risk and low‐risk groups (Table S3). Only one gene (ATP2A3) in calcium signalling pathway got a significant *P*‐value of 0.006, but it was still not more significant than the result of its corresponding subpathway (path:04020_1), which got a significant *P*‐value of 0.0004. The similar results that almost all single‐gene signatures could not distinguish the high‐risk and low‐risk groups were also observed based on the independent training set from TCGA (Table S3). Taken together, it has become obvious that the ceRNA‐mediated subpathways identified by the ce‐Subpathway method would be an effective predictor of survival outcome in cancer patients and greatly improve prognostic capabilities.

## 4 DISCUSSION

The integrative analysis of ceRNAs and DE genes at the pathway structure level will help to locate and evaluate key ceRNA‐mediated functional subpathways. We developed the ce‐Subpathway approach, which integrated ceRNAs and DE genes relevant to some given condition into pathways and identified ceRNA‐mediated functional subpathways via ceRNA interactions within pathway topologies. The algorithm has been developed as a freely available R‐based tool, which can be applicable to multiple species and various studying conditions (disease/non‐disease). In this study, the ce‐Subpathway method was firstly applied to human PAH and MI data sets, respectively. The results showed that most of the pathways identified by ce‐Subpathway were well reported to be highly associated with the corresponding diseases. And fewer pathways were not reported to be associated with PAH by curated literatures using ce‐Subpathway than the other methods. Compared with the entire pathway identification methods, ce‐Subpathway could not only locate key subpathways associated with the given condition but also identify key regions representative of entire pathways. These key subpathways contained fewer genes than entire pathways, allowing researchers to use alternative low‐throughput technologies to confirm the local subpathway regions related to specific condition. Compared with the non‐ceRNA‐mediated pathway identification methods, ce‐Subpathway was able to identify some additional ceRNA‐mediated functional subpathways. These subpathway regions contained stronger ceRNA interactions, which played important roles in evaluating the influence of competing endogenous mechanism on key subpathway identification.

This study focused on identifying subpathways by setting the threshold ω between key nodes. The threshold ω could indirectly influence the size of the located subpathway. As ω decreased, the size of the subpathway would increase because lenient distance similarity tended to merge more nodes into the same subpathway. Key nodes within entire pathways would also tend more to be added to the located subpathways. The limitations of current ceRNA identification strategy mean that there may be some false‐positive results in the ceRNA interactions used in the present study, though this pipeline is popular and has been widely applied in many ceRNA researches. Thus, we validated the reliability of ceRNA interactions based on the theory that the ceRNA interaction pairs were positively correlated. The famous ceRNA PTEN was found in the significant ceRNA‐mediated subpathways. Using the GSEA method, PTEN‐related ceRNAs showed expression down‐regulated with a cut‐off of FDR adjusted *P* < 0.05 when PTEN was knockdown. These results suggested the reliability of both ceRNA interactions used in the study and the ceRNA‐mediated subpathway identification method. Survival analysis was an effective way to test the clinical application ability of the ceRNA‐mediated subpathways. With the genes in each significant subpathway as a multi‐gene signature, the same six of eight subpathways were found to significantly distinguish patients of high‐risk and low‐risk groups in two independent training sets. More importantly, when every gene of the six common subpathways was as a single‐gene signature for survival analysis, almost all single‐gene signatures could not distinguish patients of high‐risk and low‐risk group in the same two independent training sets. Therefore, the overall discriminatory powers of the ceRNA‐mediated subpathways were further illustrated.

In the recent years, some studies have connected ceRNAs with pathways, but they usually annotated the genes associated with ceRNAs into some given pathways and got some simple results of pathway annotation. These studies did not consider the joint power of ceRNAs/DE genes and pathway topology. They also lacked systematic algorithm or available software for subpathway identification. The ce‐Subpathway method seemed to be the first implementation of ceRNA interactions within the pathways for the identification of ceRNA‐mediated functional subpathways effectively. Still, the ce‐Subpathway method has some limitations. The benchmarks used throughout to compare the performances of different pathway identification methods may be biased. However, we know that the gold standards for expected pathways associated with diseases are not clearly defined at present. We suggest that multiple integrative methods for pathway identification might need to be used in further research.

## CONFLICT OF INTERESTS

The authors declare that they have no competing financial interests.
